# Pilot deployment of a community health care worker in distributing and offering the COVID-19 AgRDT in Tanzania

**DOI:** 10.1038/s41598-024-62379-3

**Published:** 2024-05-22

**Authors:** Mwifadhi Mrisho, Grace Mwangoka, Ali M. Ali, Abdallah Mkopi, Muhidin K. Mahende, Silas Temu, Hajirani M. Msuya, Paul E. Kazyoba, Gumi Abdallah, Michael Mihayo, Omar Juma, Ali Hamad, Said Jongo, Omar Lweno, Anneth Tumbo, Sarah Mswata, Kamaka R. Kassim, Rogath Kishimba, Hussein Haruna, Hellen Kassa, Ntuli Kapologwe, Mohammed Rashid, Salim Abdulla

**Affiliations:** 1 Ifakara Health Institute (IHI), Kiko Avenue, Off Bagamoyo Road, Mikocheni, P O Box 78373, Dar es Salaam, Tanzania; 2 National Institute for Medical Research (NIMR), P O Box 9653, Dar es Salaam, Tanzania; 3 Ministry of Health (MoH), P. O. Box 743, Dodoma, Tanzania; 4 Department of Health, Social Welfare and Nutrition Services, President’s Office Regional Administration and Local Government (PORALG), P.O Box 1923, Dodoma, Tanzania; 5 FIND|Campus Biotech, Chemin des Mines 9, 1202 Geneva, Switzerland

**Keywords:** Diseases, Medical research

## Abstract

A pilot implementation of the rapid diagnostic test program was performed to collect evidence of the feasibility, acceptability, and uptake of the COVID-19 AgRDT in Tanzania. We conducted a prospective cross-sectional study in the community to provide quantitative details of the pilot implementation of the antigen rapid diagnostic test (AgRDT) in Tanzania. This study was undertaken between March 2022 and September 2022. The pilot was implemented by distributing and offering test kits to people suspected of having COVID-19 in Dar es Salaam through community health workers. A total of 1039 participants consented to participate in the survey. All the participants reported having heard about the disease. The radio was the main source (93.2%) of information on COVID-19. With regard to prevention measures, approximately 930 (89.5%) of the respondents thought that COVID-19 could be prevented. Approximately 1035 (99.6%) participants reported that they were willing to have a COVID-19 AgRDT test and wait for 20 min for the results. With regard to the participants’ opinions on the AgRDT device, the majority 907 (87.3%) felt comfortable with the test, and 1,029 (99.0%) were very likely to recommend the AgRDT test to their friends. The majority of participants 848 (83.1%) mentioned that they would be willing to pay for the test if it was not available for free. The results suggest overall good acceptance of the COVID-19 AgRDT test. It is evident that the use of trained community healthcare workers allows easy screening of all possible suspects and helps them receive early treatment.

## Introduction

The World Health Organization (WHO) recommends that all individuals suspected of being infected with COVID-19 be tested, and “*to effectively stop the spread of COVID-19, it is necessary to rapidly detect all positive cases of COVID-19 for isolation, treatment and implementation of public health control measures based on national protocols*”^1^. Rapid and accurate detection of COVID-19 infection is important for preventing the spread of infection during the COVID-19 pandemic and deaths associated with it^2,3^. Several programs on COVID-19 have been implemented during the COVID-19 era in developed and low- and middle-income countries (LMICs)^4^.

In Denmark, individuals aged 18 years or older were invited to participate in a study in which nasopharyngeal swabs were examined by the standard qCOVID-19 AgRDT test^2^. Furthermore, in the UK, real-time tracking of self-reported symptoms was performed to predict potential COVID-19^5^. The use of rapid tests in community testing in the Liverpool in the UK has led to a reduction in COVID-19 cases^6^. However, there are recommendations for additional evidence on this approach^7^. These findings have favoured the use of AgRDT in the community, particularly in resource-constrained settings such as Tanzania^6,8^.

The high running costs needed for well-equipped facilities and trained personnel^9,10^ limit the broad utilization of the standard qPCR test in resource-limited settings. Antigen-based rapid diagnostic tests (AgRDT) are advantageous because they detect active infections, especially those that can be transmitted to others^11^. These devices are simple to use, require minimal technical expertise and can be easily deployed in different settings^8,12^.

In busy health facilities, the Ag-RDT can be used to test participants in 15–20 min, whereas participants who present with symptoms consistent with COVID-19 must wait more than 20 h for qPCR test results^13^. Knowing the status of Covid-19 among these individuals may help stop the spread of Covid-19 and associated deaths^3^. These approaches will expand the scope and coverage of COVID-19 diagnosis in health facilities and in communities. One potential solution to improve access is to take services closer to communities by training local community health workers (CHWs) in the provision of diagnostic and treatment services^14,15^. CHWs can be equipped with AgRDT to expand COVID-19 testing services to benefit vulnerable populations in hard-to-reach areas. CHWs provide valuable health information and guidance to the public, as they are frequently the community's first and only point of contact, particularly for pregnant women, newborns, and children under the age of five^16^. Sociocultural constructs and knowledge of health and illness play significant roles in the diagnosis and treatment of infectious diseases^17^. Furthermore, knowledge and sociocultural constructs play a significant role in correct adaptation and use of AgRDT in the local context^18^.

Tanzania has a long history of involving CHWs in public health intervention projects^19^. Understanding the experiences and perceptions of the community regarding COVID-19 case detection may help target AgRDT interventions at the community level. Despite the promise that RDTs have shown promise in targeting COVID-19, their broader development and uptake in healthcare facilities and communities may be limited by a number of issues, including acceptability, technical shortcomings, sociocultural issues, and regulatory and economic issues, which might slow their distribution and adoption at the national and community levels^20^. Furthermore, published evidence on the performance of these RDTs thus far shows that most studies have focused on diagnostic accuracy rather than knowledge, usability and adaptation of AgRDT and were carried out in high- and middle-income countries early affected by the COVID-19 pandemic, while limited experience has existed in sub-Saharan Africa^21^. On this basis, we propose assessing the understanding, sociocultural issues, acceptability and promoters of access to AgRDT in communities. Furthermore, an assessment of the acceptance of the Ministry of Health (MoH) established algorithm for the selection of community members offered the test was also performed during this study.

## Materials and methods

### Study design

We conducted a prospective cross-sectional study in the community to provide quantitative details of the pilot implementation of the AgRDT in Tanzania. This study was undertaken between March 2022 and September 2022. The pilot was implemented by distributing and offering test kits to people suspected of having COVID-19 in Dar es Salaam through community health workers. Using a national standard testing algorithm, eligible individuals were tested, and the results were documented. Furthermore, for the evaluation of the performance of the Ministry of Health screening algorithm for testing, several other individuals lacking eligibility for testing were also offered the test for validation purposes. Our evaluations were based on the number of people who received the test kits; moreover, we explored the perceptions and acceptability of AgRDT as an enabler or inhibitor of COVID-19 deployment in Tanzania. A proof of acceptability and feasibility of using the diagnostic test was an important pre-requisite before the Government of Tanzania through the Ministry of Health authorize its use in fighting the COVID-19 pandemic. The prevalence of the AgRDT positive test was the main outcome of the main operational research; but for this sub-study the main outcome was the feasibility and acceptability.

### Institution review board statement

All the research procedures were conducted in accordance with Tanzania National laws and the guidelines of the Ethical committees and the regulatory authority^22^. The study received approval from IHI-ethics, with approval number IHI/IRB/No: 55 and the National Ethics Committee (NatHREC) NIMR/HQ/R8a/Vol.IX/3883. Written informed consent forms were sought for those individuals who participated in the evaluations that were conducted as part of the program. The consent forms outlined the proposed assessment, including the rationale, objectives, characteristics of those who participated and assessment procedures.

### Study setting

Dar es Salaam remains a major city in Tanzania and a centre of business, industry, commerce and banking activities after the government decided to move its capital to Dodoma. Administratively, Dar es Salaam has a regional administration headed by the Dar es Salaam Regional Commissioner. It also has a city council administration headed by the mayor of Dar es Salaam. The city is divided into five municipalities, namely, Ilala, Kinondoni, Ubungo, Kigamboni and Temeke, as detailed elsewhere^23^. In an effort to address the pandemic, this program aimed to cover all municipalities in Dar Es Salaam. However, due to scarcity resources, we recruited and distributed the AgRDT kits in three Municipalities only– Ilala, Kinondoni, and Temeke to capture urban, semi-urban and rural characteristics respectively.

In brief, we selected wards with at least a population of 10,000 people in Temeke Municipality, namely, Buza, Chamazi, Mbagala kuu and Sandali, while in Kinondoni, we selected the Kigogo, Kijitonyama, Msasani and Ndugumbi wards. In the Ilala Municipality, we selected the Mnazi Mmoja, Pugu, Tabata and Kivule wards.

### Community engagement

A series of open meetings were held at higher and lower levels of the local government structure as well as in the selected communities. The study team presented the proposal to the National COVID-19 Response Committee twice to fine-tune the protocol into the local context. In addition, a number of local stakeholders, such as counsellors, ward leaders and street leaders, were engaged to create demand for AgRDT testing in the selected areas in Dar es Salaam. These meetings helped to establish buy in the program implementation activities, as well as to structure a range of materials to support the development of information and education and communication (IEC) campaigns. In these meetings, the communities were informed about the availability of AgRDT Kits in the health facilities and through the CHWs for COVID-19 detection. Mobile numbers of CHWs were provided/displayed in the selected communities to request AgRDT testing from the CHWs. The campaign materials were pilot tested within the study team and the stakeholders before printing and distribution to the communities and clinic outlets. The content of the materials included information on the aim of the study, targeted group, risk and benefit for participation, how to join in the study, who is implementing the study. The material, had a space for putting a mobile number of the CHW. These materials were put into the offices of the ward secretaries as well as local health facilities.

In each municipality of Dar es Salaam region, network CHWs were identified. After consultations with the three municipality health authorities, 4 health facilities (lower level) were selected in each municipality as deployment points for the AgRDT kits. In total, 12 health facilities were identified as deployment sites. A research assistant (RA) was positioned at each municipality to supervise operational research activities and CHWs in the selected areas. Likewise, after consultations with health facility staff and village/street leaders, 5 CHWs were selected among the existing CHWs in the selected health facilities. The selected CHWs distributed and supervised the testing in the community passively and actively. With passive distribution and testing, the community members requested AgRDT kits from the CHWs through mobile phones. With active distribution, the CHWs were moving around the households to find the cases.

The selection criteria of the CHWs were based on sex balance, residential area and the ability to read, write and keep records. One-day training with CHWs was conducted by medical technologists from the national laboratory at the Ifakara Health Institute. The CHWs were trained on how to recognize symptoms of COVID-19 as per the MoH guidelines. In addition, the CHW were trained on how to perform and interpret AgRDT according to the manufacturer's instructions and simplified pictorial instructions (AgRDT job aid).

### Recruitment procedure at the community

Although the prevalence of the positive test was not one of the outcomes in this study, it was primarily driven by the projected burden of COVID-19 in the selected community and this was to be compared to the proportion of COVID-19 positive person identified in the health facility. It was assumed that the proportion of COVID-19 positive samples would be 2% in the health facility and 1% in the community. To estimate the 1% prevalence (+ /– 1%) at 97% confidence level a total number 1000 individuals (including 9% incomplete information) to be offered the test was to be sampled. As the estimate of the proportion of positive tests in those that were not selected to be offered the test was expected to be very low a convenient sample of 1000 were to be recruited in this group. The percentage of refusals for the test was assumed to be 20% and hence the stated sample was adequate to correctly estimate the proportion of refusals.

All identified adults older than 18 years were subjected to AgRDT tests, and the results were obtained during implementation in the community using the established clinical algorithm of the MoH. A convenient sampling of those who agreed to participate in the AgRDT was given study information and asked for written consent to participate in a prospective cross-sectional study during evaluation. According to this study, we defined uptake as the willingness to have a Covid-19 rapid test and wait the results for 20 min and recommend AgRDT test to others. In addition, feasibility was defined as perception or experience with regards to the use of AgRDT devices. Likewise, acceptability was defined as the likelihood of recommending AgRDT test to others or willingness to purchase for a COVID-19 test kit if it’s not available for free. For the purpose of this study, we therefore designed the tool to capture the information related to feasibility and acceptability accordingly.

All the consented participants had their sociodemographic information, such as sex, age, occupation, educational level, COVID-19 testing history, COVID-19 symptoms, medical history, signs and symptoms documented. Questions related to knowledge, attitudes, practices as well as feasibility, acceptability and uptake of AgRDT were also captured.

### Data collection, management, storage and analysis

All the data were collected using semi structured questionnaires loaded on tablets/mobile phones, except for the consent forms, which were completed on paper. We trained RAs and CHWs on how to capture, enter and submit community data to the IHI server through tablets, computers or smartphones loaded with the open data kit (ODK)-open mobile data collection platform.

We used descriptive statistics to summarize the quantitative data; categorical data were summarized using frequency counts and percentages, whereas continuous variables were summarized with means and standard deviations. For knowledge, a question with multiple choices was given a score of one for each choice, and an average was calculated. An average score of 50% or above was arbitrarily considered to indicate knowledge of that question. Disease symptoms, disease spread and risk group were scored^24^. Data analysis was performed with Stata 15 standard editions (StataCorp, Texas, USA)^25^.

## Results

### Demographic characteristics of the participants

A total of 1039 participants consented to participate in the cross-sectional survey. More than half of the participants were female. The mean (standard deviation [SD]) age of the participants was 36 (13.2) years, and 66.1% were aged between 18 and 40 years. The majority of the participants had completed primary education followed by secondary education. More than half of the participants were married or cohabiting. Most of the surveyed participants were self-employed and engaged mainly in business. The characteristics of the study participants are presented in Table 1.

**Table 1:** Demographic information.

Variable	Eligible*	Non-eligible	Total
Number of participants surveyed	684	355	1039
Gender			
Male	303 (44.3)	143 (40.3)	446 (42.9)
Female	381 (55.7)	212 (59.7)	593 (57.1)
Age–mean ± SD (years)	36.9 ± 13.5	34.9 ± 12.5	36.2 ± 13.2
18≤ 40	439 (64.2)	248 (69.9)	687 (66.1)
≥ 40	245 (35.8)	107 (30.1)	352 (33.9)
District			
Ilala	255 (37.4)	92 (25.9)	347 (33.4)
Kinondoni	253 (36.9)	112 (31.6)	365 (35.1)
Temeke	176 (25.7)	151 (42.5)	327 (31.5)
Marital status			
Unmarried	262 (38.3)	126 (35.5)	388 (37.3)
Married/cohabited	363 (53.1)	200 (56.3)	563 (54.2)
Widowed	29 (4.2)	15 (4.3)	44 (4.2)
Divorced	30 (4.4)	14 (3.9)	44 (4.2)
Highest educational level attained			
None	14 (2.1)	17 (4.8)	31 (3)
Primary	303 (44.3)	170 (47.9)	473 (45.5)
Secondary	276 (40.3)	140 (39.4)	416 (40)
College and above	91 (13.3)	28 (7.9)	119 (11.5)
Employment status			
Student	28 (4.1)	14 (3.9)	42 (4)
Employed	58 (8.5)	34 (9.6)	92 (8.9)
Unemployed	127 (18.6)	83 (23.4)	210 (20.2)
Self employed	434 (63.5)	214 (60.3)	648 (62.4)
Farming	18 (2.6)	1 (0.3)	19 (1.8)
Health/medical	19 (2.7)	9 (2.5)	28 (2.7)
Occupation/trade			
Transport	31 (5.6)	14 (5.2)	45 (4.3)
Education	31 (5.6)	22 (8.1)	53 (5.1)
Business	361 (64.8)	180 (66.2)	541 (52.1)
Health/medical	30 (5.4)	8 (2.9)	38 (3.7)
Other	10 (1.8)	13 (4.8)	23 (2.2)

Values are reported as n (%).

*An adapted Tanzania Ministry of Health defined algorithm was used to decide persons in the community who are most likely to benefit from the AgRDT testing to be offered the test. The known ten symptoms for Covid-19 infection are; fever, persistent cough, fatigue, shortness of breath, diarrhoea, delirium, skipped meals, abdominal pain, chest pain, and hoarse voice [García-Fiñana, M., & Buchan, I. E.^6^] https://www.covidlawlab.org/item/criteria-for-covid-19-testing/.

#### Knowledge, attitudes and practice

**Figure 1:**
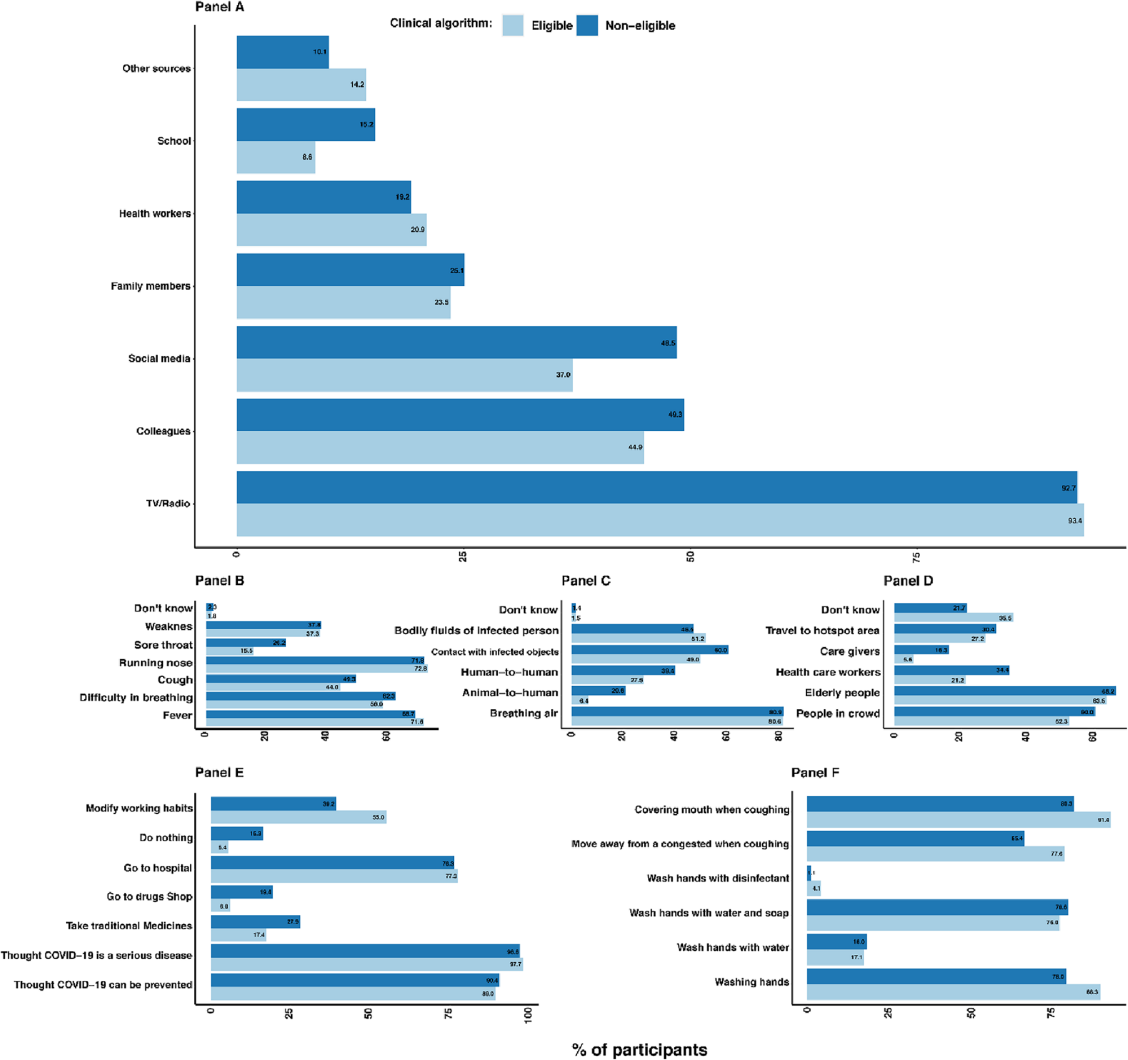
Source of information, knowledge, attitudes, and practices related to COVID-19.

This evaluation documented knowledge, attitudes, and practices related to COVID-19 and is presented in Fig. 1. All the participants reported having heard about the disease. The radio was the main source of information on COVID-19. In addition, 46.4% of the participants heard from their colleagues, 40.9% heard from social media, and only 24.1% heard from family members. Participants at a younger age (18–40 years) heard more about COVID-19 disease via social media than did those > 40 years, p value < 0.001.

Participants were asked questions related to COVID-19 to test their knowledge. Six hundred and fifty eighty participants (63.3%) knew about the signs and symptoms related to COVID-19, 422 (40.6%) knew how the disease was spread out, and 282 (27.1%) knew about the risk group. Runny nose, fever and difficulty breathing were mentioned as the most common signs/symptoms of CODIVD-19 by 72.5%, 70.6% and 59.5%, respectively, of the participants. Cough was mentioned by only 45.8% of the participants. Breathing infected air was mentioned by 80.7% of participants as the source of COVID-19 spread, while only 11.3% of the participants mentioned animal-to-human infection. A total of 669 elderly people (64.4%) and 571 people in crowded places (55.0%) were mentioned as being at higher risk.

With regard to prevention measures, approximately 930 (89.5%) of the respondents thought that COVID-19 could be prevented. Likewise, 1011 (97.3%) thought that the disease was serious, 327 (31.5%) participants thought that COVID-19 could be treated, and only 240 (23.1%) participants did not know if the disease was treatable. The majority of the participants 881 (84.8%) mentioned having washed their hands. With regard to how the participants washed their hands, 799 (76.9%) washed their hands with water and soap, 181 (17.2%) washed their hands with water, and only 32 (3.1%) of the participants mentioned having washed their hands with disinfectant (Fig. 1).

The participants were also asked how often they moved away from a congested place when coughing or sneezing. This question wanted to capture how COVID-19 measures were being implemented in the community. Approximately 763 (73.4%) of the participants reported having moved away from the congestion when coughing or sneezing. With regard to the use of facemasks, 755 (72.7%) of the participants reported using facemasks. Participants were asked how they would rate their fear of contracting COVID-19. Eight hundred and thirteen participants (78.2%) mentioned that they had fear of COVID-19. With regard to the willingness to test for COVID-19, more than 1,020 (98%) participants were willing to test for COVID-19 to protect their loved ones.

#### Perception toward AgRDT testing

Participants were asked about their experience and perceptions of using AgRDT. One thousand thirty-five participants (99.6%) reported that they were willing to complete the COVID-19 AgRDT test and wait for 20 min for the results. With regard to the participants’ opinions on the AgRDT device, the majority 907 (87.3%) felt comfortable with the test, and 1029 (99.0%) were very likely to recommend the AgRDT test to their friends. It was also reported that the test took short time to use, and it was user friendly. The participants were also asked about their willingness to pay for a COVID-19 test kit if it was unavailable for free. The majority of participants 848 (83.1%) mentioned that they would be willing to pay for the test if it was not available for free. On average, the participants were willing to pay approximately 3000 TSHs for AgRDT kits (equivalent to 1.2$). The perception towards AgRDT testing are presented in Table 2.

**Table 2:** AgRDT Experiences and perception.

Variable	Clinical algorithm		Total
	Eligible	Not eligible	
Willingness to have a Covid-19 rapid test and wait the results for 20 min	683 (99.9)	352 (99.2)	1035 (99.6)
Participant’s opinion with regards to the device use by the help of CHW			
Took shot time to use	366 (53.5)	221 (62.3)	587 (56.5)
User friendly	280 (40.9)	128 (36.1)	408 (39.3)
Other	38 (5.6)	6 (1.7)	44 (4.2)
Feel about the testing process itself			
Comfortable	586 (85.7)	321 (90.4)	907 (87.3)
Experienced some pain	61 (8.9)	18 (5.1)	79 (7.6)
Uncomfortable	14 (2.1)	11 (3.1)	25 (2.4)
Other	23 (3.4)	5 (1.4)	28 (2.7)
Willing to recommend AgRDT test to others:	679 (99.3)	350 (98.6)	1029 (99)
Pay for a COVID-19 test kit if it’s not available for free	576 (85)	272 (79.5)	848 (83.1)

Values are reported as n (%).

Seven hundred and fifty-six (72.8%) of the participants reported their willingness to test for COVID-19 even though they had been vaccinated. When asked about the reasons for testing, the majority 904 (87.0%) mentioned that they would perform a COVID-19 test for self-protection, 315 (30.3%) would test to protect others or to stop transmission, and 170 (16.4%) would test for travel purposes.

### Perception of the COVID-19 vaccine

The study also aimed to determine participants’ perceptions of the COVID-19 vaccine. Seventy-three percent of the respondents received the COVID-19 vaccine. Of those who were vaccinated, 756 (99.3%) reported that they were willing to test for COVID-19 even though they had been vaccinated. The majority of the participants mentioned that it was necessary to receive the vaccine to protect themselves—377 (36.3%) to protect others—while 170 (16.4%) to stop transmission in the community and 315 (30.3%) for travel purposes (refer Table 3 on the perception of the COVID-19 vaccine).

**Table 3:** Perception toward COVDI-19 vaccine.

Variable	Clinical algorithm		Total
	Eligible	Not eligible	
Perception toward COVDI-19 vaccine			
Received a COVID-19 vaccination	512 (74.9)	249 (70.1)	761 (73.2)
Supposed to return for the second dose	79 (15.4)	30 (12.1)	109 (14.3)
Received the second dose	54 (68.4)	21 (70)	75 (68.8)
Willingness to test for a COVID-19 in addition to be vaccinated	92 (53.5)	48 (45.3)	140 (50.4)
Willingness to get COVID-19 vaccine	510 (74.6)	246 (69.3)	756 (72.8)
Necessary to test COVID-19 in order to			
Self-protection	601 (87.9)	303 (85.4)	904 (87)
Protect other	212 (31)	165 (46.5)	377 (36.3)
Stop transmission in your community	93 (13.6)	77 (21.7)	170 (16.4)
For Travel purpose	207 (30.3)	108 (30.4)	315 (30.3)

Values are reported as n (%).

## Discussion

This pilot program collected evidence of the feasibility, acceptability, and, more importantly, uptake of AgRDT at the community level. The results suggest overall good acceptance of the COVID-19 AgRDT test. The introduction of CHW testing increased the scope of testing by reaching even those who were not reached by the COVID-19 vaccination campaign.

This study assessed for the first time the community acceptability of COVID-19 AgRDT among community health workers. Therefore, it has been demonstrated that the CHW can be trained and test community members with COVID-19 using AgRDT, as shown elsewhere^26^. The data suggest that the majority of the participants had adequate knowledge associated with COVID-19. Despite the adequacy of their knowledge, attitudes and practices, some participants could not understand how COVID-19 is spreading, as documented elsewhere^27^. This requires additional effort to increase awareness of preventive practices against the transmission of COVID-19. Participants’ major sources of COVID-19 information were radio, TV, colleagues, social media and family members. This finding is similar to what was reported elsewhere^28–33^.

Interestingly, participants at a younger age (18–40 years) were more likely to obtain information from social media than were those > 40 years. The provision of more education at the community level on the importance of COVID-19 testing, especially in the community, will increase the uptake of the test, as confirmed by the results and other studies^34^.

As was reported by the majority of participants, experience using the AgRDT was good. Almost all participants reported that they were willing to complete the COVID-19 AgRDT test and wait for 20 min for the results. This finding is similar to what was reported elsewhere by Nwagbara et al.^27^. Those who used AgRDT kits felt comfortable with the test, and all participants were very likely to recommend the AgRDT test to their friends. This study revealed that the majority of participants mentioned having a runny nose, fever or difficulty breathing as the most common symptoms of COVID-19. This finding is similar to what was reported from other parts of the world^27^.

The delivery of nasal Ag-RDT through trained CHWs has broadened access to diagnostic tools in the community^35^. Our findings are consistent with those of a study conducted in South Africa in which the CHW increased the turnaround time for test results, as laboratories were overwhelmed during the COVID-19 era^26,35,36^.

The lesson learned is similar to what was reported by David & Mash^36^ for tackling epidemics in communities^36^. This study is consistent with other studies on the loyalty of CHWs in that lower-level health workers are more likely to comply with guidelines than more experienced or high-cadre health workers are^37^.

In the era of the COVID-19 pandemic, several LMIC countries established community health worker programmes to deliver diagnostic testing, treatment, immunization and contact tracing^38^. It is for this reason that our study has pioneered to be among the first countries in the LMICS to demonstrate that the CHWs can deliver AgRDT in the community. The implementation of the study using available diagnostic devices and platforms has made it easier for the health system to adapt and handle public health issues such as the COVID-19 pandemic.

Our pilot study generated evidence on the feasibility of the operationalization of AgRDT through the CHW strategy in the community. The successful implementation of this work may have been related to the supervision of the study through the RAs who were placed in each municipality.

The deployment of AgRDT in the community has provided an opportunity for building a case on the necessity and feasibility of conducting active surveillance of the pandemic as well as simplifying the detection of new cases among suspected individuals. This undertaking has excellently taken us through new milestones in understanding alternative ways to deploy new interventions, especially pandemics such as COVID-19.

We also identified key issues that could affect participants’ decisions to use the AgRDT. This approach is expected to support the development of an evidence-based AgRDT scale-up strategy to ensure optimal acceptance of AgRDT among the Tanzanian population. The provision of AgRDT by CHWs is likely to be acceptable to community members given that CHWs are well accepted, trained, supervised and given logistical support. The gender balance and level of formal education may be considered when CHWs are selected for use in programmes deploying AgRDTs.

### Limitations

Study limitations: First, our study was conducted during a period of low COVID-19 transmission in the community, and the sample size may not be sufficient due to limitations in the length and resources of the study. Second, our study lacked sampling fame and included a convenient sampling of the general public in the selected communities who consented to the evaluation of AgRDT. Selection bias is thus likely to have happened. Despite the limitations of this study, it helped to improve the critical gap in access to AgRDT through CHWs, which increased the number of people tested from low-resource communities; this finding is compatible with Sania et al.^26^ and David & Mash^26,36^. With the availability of results within twenty minutes, people in these communities were keen to be tested with the guarantee of confidentiality and trusted personnel in their own community. This finding is similar to what was reported in Bangladesh^26^. Finally, the CHWs were useful and cost effective in implementing this program, and there is a need to incorporate this program in the framework of primary health care for Tanzania and beyond.

Reporting emerging and re-emerging pathogens, such as influenza-like illnesses, will further promote the use of the test and reporting. In this pilot study, we assessed the extent of acceptance of AgRDT among participants through CHWs at the community level.

## Conclusion

AgRDT deployment for detection and decision-making in Tanzania is a feasible, acceptable, and cost-effective approach in the fight against COVID-19. It is evident that the use of trained community healthcare workers allows easy screening of all possible suspects and helps them receive early treatment. CHWs are well trusted by community members; hence, when trained, they can easily perform the testing and capture the data electronically. Our general recommendations are for constant knowledge provision and awareness to the general community on the existence of the disease but on the need for continued fight until the disease is declared extinct or never a threat to human lives. This will be achieved by continued surveillance of the disease and prompt measures taken whenever possible.

## Data Availability

Where possible, dataset used may be made available to facilitate editorial and peer review purposes from the corresponding author.

## References

[1] World Health Organization, *SARS-CoV-2 antigen-detecting rapid diagnostic tests: An implementation guide* (2020).

[2] Jakobsen KK (2021). Accuracy and cost description of rapid antigen test compared with reverse transcriptase-polymerase chain reaction for SARS-CoV-2 detection. Dan Med. J..

[3] Valentine-Graves M (2020). At-home self-collection of saliva, oropharyngeal swabs and dried blood spots for SARS-CoV-2 diagnosis and serology: Postcollection acceptability of specimen collection process and patient confidence in specimens. PLoS one.

[4] Abdullahi L (2020). Community interventions in Low—And Middle-Income Countries to inform COVID-19 control implementation decisions in Kenya: A rapid systematic review. PLoS One.

[5] Menni C (2020). Widespread smell testing for COVID-19 has limited application–Authors' reply. Lancet.

[6] García-Fiñana M, Buchan IE (2021). Rapid antigen testing in COVID-19 responses. Science.

[7] Wise J (2021). Covid-19: Rapid Testing Cuts Cases in Pilot But Questions Remain Over use of Lateral Flow Tests.

[8] Olalekan A (2020). COVID-19 rapid diagnostic test could contain transmission in low-and middle-income countries. Afr. J. Lab. Med..

[9] Jayamohan H (2021). SARS-CoV-2 pandemic: A review of molecular diagnostic tools including sample collection and commercial response with associated advantages and limitations. Anal. Bioanal. Chem..

[10] Sidiq Z (2020). Benefits and limitations of serological assays in COVID-19 infection. Indian J. Tuberc..

[11] Omollo MC (2022). Health workers’ perspective on the feasibility and acceptability of the introduction of AgRDT for COVID-19 in Kisumu County, Western Kenya. medRxiv.

[12] Kumar R (2020). COVID-19 diagnostic approaches: Different roads to the same destination. Virusdisease.

[13] Peeling RW, Olliaro P (2021). Rolling out COVID-19 antigen rapid diagnostic tests: the time is now. Lancet Infect. Dis..

[14] Haines A (2007). Achieving child survival goals: potential contribution of community health workers. Lancet.

[15] Paintain LS (2014). Community health workers and stand-alone or integrated case management of malaria: A systematic literature review. Am. J. Trop. Med. Hygiene.

[16] Feroz AS, Khoja A, Saleem S (2021). Equipping community health workers with digital tools for pandemic response in LMICs. Arch. Public Health.

[17] Bayeh R, Yampolsky MA, Ryder AG (2021). The social lives of infectious diseases: Why culture matters to COVID-19. Front. Psychol..

[18] Jacobs J (2020). Implementing COVID-19 (SARS-CoV-2) rapid diagnostic tests in Sub-Saharan Africa: A review. Front. Med..

[19] Mubi M (2013). Malaria diagnosis and treatment practices following introduction of rapid diagnostic tests in Kibaha District, Coast Region, Tanzania. Malar. J..

[20] Miller E, Sikes HD (2015). Addressing barriers to the development and adoption of rapid diagnostic tests in global health. Nanobiomedicine.

[21] Bastos ML (2020). Diagnostic accuracy of serological tests for covid-19: systematic review and meta-analysis. BMJ.

[22] Committee NHRE (2001). Guidelines on Ethics for Health Research in Tanzania.

[23] Todd G (2019). City Profile: Dar es Salaam, Tanzania. Environ. Urban. ASIA.

[24] Bendel RB, Afifi AA (1977). Comparison of stopping rules in forward “stepwise” regression. J. Am. Stat. Assoc..

[25] StataCorp L (2017). Stata Spatial Autoregressive Models Reference Manual.

[26] Sania A (2022). Rapid antigen testing by community health workers for detection of SARS-CoV-2 in Dhaka, Bangladesh: A cross-sectional study. BMJ Open.

[27] Nwagbara UI (2021). Knowledge, attitude, perception, and preventative practices towards COVID-19 in sub-Saharan Africa: A scoping review. PLoS One.

[28] Olum R (2020). Coronavirus disease-2019: Knowledge, attitude, and practices of health care workers at Makerere University Teaching Hospitals Uganda. Front. Public Health.

[29] Akalu Y, Ayelign B, Molla M (2020). Knowledge, attitude and practice towards COVID-19 among chronic disease patients at Addis Zemen hospital, Northwest Ethiopia. Infect. Drug Resist..

[30] Adhena G, Hidru HD (2020). Knowledge, attitude, and practice of high-risk age groups to Coronavirus Disease-19 prevention and control in Korem District, Tigray, Ethiopia: Cross-sectional study. Infect. Drug Resist..

[31] Asemahagn MA (2020). Factors determining the knowledge and prevention practice of healthcare workers towards COVID-19 in Amhara region, Ethiopia: A cross-sectional survey. Trop. Med. Health.

[32] Asmelash D (2020). Knowledge, attitudes and practices toward prevention and early detection of COVID-19 and associated factors among religious clerics and traditional healers in Gondar Town, Northwest Ethiopia: A Community-Based Study. Risk Manag. Healthc. Policy.

[33] Girma S, Alenko A, Agenagnew L (2020). Knowledge and precautionary behavioral practice toward COVID-19 among health professionals working in public university hospitals in Ethiopia: A web-based survey. Risk Manag. Healthc. Policy.

[34] Moola S (2021). A rapid review of evidence on the determinants of and strategies for COVID-19 vaccine acceptance in low-and middle-income countries. J. Glob. Health.

[35] Organization, W.H., Selection of essential in vitro diagnostics at country level: using the WHO model list of essential in vitro diagnostics to develop and update a national list of essential in vitro diagnostics (2021).

[36] David N, Mash R (2020). Community-based screening and testing for Coronavirus in Cape Town, South Africa. Afr. J. Primary Health Care Fam. Med..

[37] Zurovac D (2004). Predictors of the quality of health worker treatment practices for uncomplicated malaria at government health facilities in Kenya. Int. J. Epidemiol..

[38] Bhaumik S (2020). Community health workers for pandemic response: A rapid evidence synthesis. BMJ Glob. Health.

